# Blood Cathepsins on the Risk of Alzheimer’s Disease and Related Pathological Biomarkers: Results from Observational Cohort and Mendelian Randomization Study

**DOI:** 10.14283/jpad.2024.107

**Published:** 2024-06-11

**Authors:** X.-H. Qian, G.-Y. Ding, S.-Y. Chen, Xiao-li Liu, Miao Zhang, Hui-dong Tang

**Affiliations:** 1 Department of Geriatrics, Ruijin Hospital, Shanghai Jiao Tong University School of Medicine, Shanghai, China; 2 Medical Center on Aging of Ruijin Hospital, Shanghai Jiao Tong University School of Medicine, Shanghai, China; 3 Department of Neurology and Institute of Neurology, Ruijin Hospital, Shanghai Jiao Tong University School of Medicine, Shanghai, China; 4 Department of Neurology, Jiading District Central Hospital Affiliated Shanghai University of Medicine and Health Sciences, Shanghai, China; 5 Department of Neurology, Shanghai Fengxian District Central Hospital, Shanghai University of Medicine & Health Science Affiliated Sixth People’s Hospital South Campus, Shanghai, China; 6 Department of Nuclear Medicine, Ruijin Hospital, Shanghai Jiao Tong University School of Medicine, Shanghai, China

**Keywords:** Alzheimer’s disease, cathepsins, mendelian randomization, pathological features

## Abstract

**Background:**

Alzheimer’s disease (AD), the main type of dementia, involves in complex pathophysiological processes, including abnormal lysosomes function. Cathepsins are the predominant proteases responsible for the degradation of diverse substrates in the endo-lysosomal system. However, there was still a lack of systematic study on the causal association between cathepsins and AD.

**Methods:**

This study utilized Mendelian randomization (MR) to investigate the association between blood cathepsins and the risk of AD, as well as the level of amyloid-β (Aβ) and p-Tau in cerebrospinal fluid. Furthermore, an independent dataset was employed to corroborate the above result. Importantly, this study incorporated the Alzheimer’s disease Immunization and Microbiota Initiative study Cohort to further validate the alteration of blood cathepsins expression level and examine its correlation with cognitive level and plasma AD-related pathological markers.

**Results:**

Using MR method, we observed that high level of cathepsin L (CTSL) was associated with a lower risk of AD in both training and validation data. In observational cohort, we found there was decreased blood CTSL expression level in Aβ+ cognitive impaired (CI) group, compared with Aβ− cognitive unimpaired (CU) group. Correlation analysis revealed that blood CTSL expression level was negatively correlated with Mini-Mental State Examination (MMSE) and Montreal Cognitive Assessment (MoCA) score, plasma Aβ42 and Aβ42/40 level in Aβ+ CI group. Mediation analysis showed that plasma Aβ42/40 level was the key mediator in the association between blood CTSL and MMSE score in Aβ+ CI participants.

**Conclusion:**

This study revealed that blood CTSL was an important factor affecting the risk of AD, and it affected the cognitive level of AD patients through plasma Aβ42/40 level.

**Electronic Supplementary Material:**

Supplementary material is available for this article at 10.14283/jpad.2024.107 and is accessible for authorized users.

## Introduction

**A**lzheimer disease (AD), a progressive neurodegenerative disease, has affected more than 50 million people and is the seventh leading cause of death in the world (1, 2). Even worse, as the global aging process intensifies, the number of people with AD will increase year by year, and is expected to grow to 150 million patients by 2050 (1). The pathological changes of AD is characterized by the extracellular amyloid-β (Aβ) plaques deposition and intracellular neurofibrillary tangles (NFTs) formed by hyperphosphorylated tau protein (3). Although these abnormally aggregated proteins represent the main pathological features of AD, a series of studies have found that AD involves a variety of other complex pathophysiological processes, such as synaptic/neuronal degeneration, neuroinflammation, blood-brain barrier (BBB) disruption, metabolic dysregulation, etc (4, 5). More importantly, lysosomal dysfunction is one of the important pathways mediating these pathophysiological processes (6, 7). But the specific molecular mechanism is not clear.

Cathepsins, as the predominant lysosomal enzymes, play a key role in the extensive degradation of diverse substrates (8). According to their catalytic sites, the cathepsins family are grouped into three different families, including: two serine proteases (cathepsins A and G), 11 cysteine proteases (cathepsins B, C, F, H, K, L, O, S, V, W, and Z), and two aspartic proteases (cathepsins D and E) (6, 9). Cathepsins play essential role in a wide range of intra- and extracellular processes, including but not limited to antigen presentation, remodeling of the extracellular matrix, autophagy, antigen processing, regulating immune response (6, 10, 11). In recent years, a series of clinical and preclinical studies have elucidated the association and function of cathepsins with neurodegenerative diseases, especially AD (6). For example, genetic studies have confirmed that polymorphisms of cathepsin D and H are associated with the risk of AD (12, 13). In addition, abnormal expression levels of cathepsin D and B in the plasma of patients with AD has also been reported in several studies (14–16). However, the results reported by different studies are inconsistent, which may be related to confounding bias and reverse causation bias in observational studies. At the same time, there is a lack of systematic study of cathepsins on the risk of AD and related pathological features.

The mendelian randomization (MR) approach was developed to investigate causal relationships between exposures and outcomes by utilizing genetic instrumental variables (IVs) (17, 18). These genetic IVs are specifically associated with the exposures and remain unaffected by any confounding factors (17). Therefore, this study utilized MR methods to investigate the association between blood cathepsins and the risk of AD, as well as the levels of Aβ and p-Tau in cerebrospinal fluid (CSF). Furthermore, an independent AD genome-wide association data (GWAS) dataset was employed to corroborate the findings through MR analysis. Importantly, this study incorporated the Alzheimer’s disease Immunization and Microbiota Initiative study Cohort (ADIMIC) to further validate the alterations of blood cathepsin expression level and examine their correlation with cognitive level and plasma AD-related pathological markers (Aβ40, Aβ42, Aβ42/40, p-Tau181, glial fibrillary acidic protein (GFAP), and neurofilament light (NFL)).

## Method

### Study design

We used MR study based on large sample GWAS data and population-based observational cohort study to comprehensively explore the association of cathepsins with the risk of AD and related pathological features. First of all, we utilized MR method to investigate the association between blood cathepsins and the risk of AD, as well as the levels of Aβ and p-Tau in CSF. After that, an independent AD GWAS dataset was employed to corroborate the above result. In addition, this study incorporated the ADIMIC cohort to further validate the alterations of blood cathepsin expression level and examine their correlation with cognitive level and plasma AD-related pathological markers (Aβ40, Aβ42, Aβ42/40, p-Tau181, GFAP, and NFL) (Figure S1).

### MR analysis

In this research, data on the exposure of various cathepsins were gathered from a large sample of plasma proteomic GWAS data consisting of 35,559 participants (19). The outcome data came from a recent large-scale AD GWAS meta-analysis dataset from the European Alzheimer & Dementia Biobank (EADB) stage I, which included 85,934 clinically diagnosed or ‘proxy’ AD cases and 401,577 controls (20). Additionally, GWAS data from a meta-analysis on AD CSF biomarkers (Aβ42 and p-Tau) from 13,116 participants were used as outcome data (21). To validate the findings, an earlier GWAS dataset from the International Genomics of Alzheimer’s Project (IGAP) was utilized, which included 21,982 late-onset AD cases and 41,944 controls (22). All participants in these datasets were of European descent. Genetic IVs for various cathepsins and AD were extracted under the same criteria. P < 5 × 10^−8^ was selected as a statistical difference threshold of all relevant SNPs from each GWAS. Under the threshold of r^2^ < 0.001 in a 10000kb window, the PLINK clumping algorithm was used to prune for independence of SNPs in linkage disequilibrium (23). The palindromic SNPs with a minor allele requency (MAF) of <0.01 were excluded from the above instrument SNPs. The possible confounding of the exposure-outcome associated SNPs were removed according to the PhenoScanner GWAS database (http://phenoscanner.medschl.cam.ac.uk) (24). In this study, age and gender were selected as the confounding factors. Furthermore, the F statistic > 10 was set as a threshold to assess the strength of the selected genetic variants (25). The inverse-variance weighted (IVW) was applied to the standard MR analysis and verified by MR-Egger, weighted median, and weighted mode methods (26, 27). The Cochran’s Q statistic was used to evaluate the heterogeneity of IVW (28). If p-value ≥ 0.05, random effect IVW method was selected. The MR-Egger intercept test was used to estimate the potential horizontal pleiotropy of the MR results (27). If p-value < 0.05, horizontal pleiotropy was present and the result was not included in the analysis.

### Cohort study

#### Study participants

Participants in this study were recruited from the ADIMIC study. This study protocol was approved by the Review Committee Research Committee of Ruijin Hospital, Shanghai Jiao Tong University School of Medicine (approval number: 2021–46). All participants provide written informed consent prior to participation. All participants completed comprehensive neuropsychological assessments, ^18^F-florbetapir PET/MR, whole blood RNA sequencing and plasma AD biomarkers. A diagnosis of mild cognitive impairment (MCI) or AD dementia was assigned according to the Petersen criteria and the 2011 National Institute on Aging and Alzheimer’s Association (NIA-AA) diagnostic criteria, respectively (29, 30). In this study, participants with clinical diagnosis of MCI/AD dementia and Aβ+ PET were enrolled and defined as the Aβ+ cognitive impaired (CI) group. Participants with normal cognitive level and Aβ− PET were defined as the Aβ− cognitive unimpaired (CU) group.

### Cognitive assessments

All participants completed comprehensive neuropsychological assessments, including MiniMental State Examination (MMSE), Montreal Cognitive Assessment (MoCA), Clinical dementia rating (CDR), the Auditory Verbal Learning Test (AVLT), boston naming task, digital breadth test, connection test.

#### ^18^F-AV45 PET/MR imaging acquisition and visual assessment

All participants completed the ^18^F-AV45 PET/MR imaging acquisition. The standardized PET/MR imaging acquisition and visual assessment were described in our previous study (31). In brief, after 40 to 60 minutes of intravenous infusion of ^18^F-AV45 with a mean dose of 260.65 ± 40.34-MBq, MRI and PET imaging data were collected simultaneously. MRI imaging included the three-dimensional T1-magnetisation prepared-rapid gradient echo (MPRAGE), fluid-attenuated inversion recovery (FLAIR) sequences, axial two-dimensional T2, coronal T2, coronal FLAIR sequences, and ^18^F-AV45 PET images. The ^18^F-AV45 PET images were visually assessed by two experienced radiologists with nuclear medicine and radiology certificates according to the International Nuclear Medicine Consensus on the Clinical Use of Amyloid Positron Emission Tomography in AD (32).

#### Measurements of blood CTSL and AD biomarkers

Peripheral whole blood was collected from each participant and plasma was separated. Peripheral whole blood and plasma were stored at −80 °C before detection. RNA transcriptome sequencing of whole blood was performed by the Shanghai Applied Protein Technology Co., Ltd. (APTBIO, Shanghai, China). The standardized testing method was described in our previous study (31). In addition, single molecule array (Simoa) technique was used to detect the levels of Aβ40, Aβ42, GFAP, NFL and p-Tau181 in plasma. Plasma p-Tau181 was tested using the Simoa® p-Tau181 kit. Plasma Aβ42, Aβ40, GFAP, and NFL use the Simoa®N4PE Advantage kit. All procedures were standardized according to the instructions provided with the kit.

### Statistical Analysis

The two-sample MR analyses were performed by the “TwoSampleMR” and the “MR-PRESSO” packages in R software (v4.2.0). The statistical P value for causality detection was set as < 0.00625 (0.05/8) through Bonferroni method. The statistical P value for horizontal pleiotropy and heterogeneity test was set as < 0.05. The meta-analysis was performed by STATA software version 14.0. P < 0.05 was set as the statistical threshold for the meta-analysis. In ADIMIC study, the difference of continuous variable between the two groups was analyzed by two-tailed Student’s t-test. The difference of categorical variable between the two groups was analyzed by Chisquare test. Spearman analysis was used for correlation analysis. The mediation analysis was performed by SPSS 22.0. P < 0.05 was set as a statistical difference.

## Result

### Two-sample MR analysis defined blood cathepsins on the risk of AD and related pathological features

**Figure 1:**
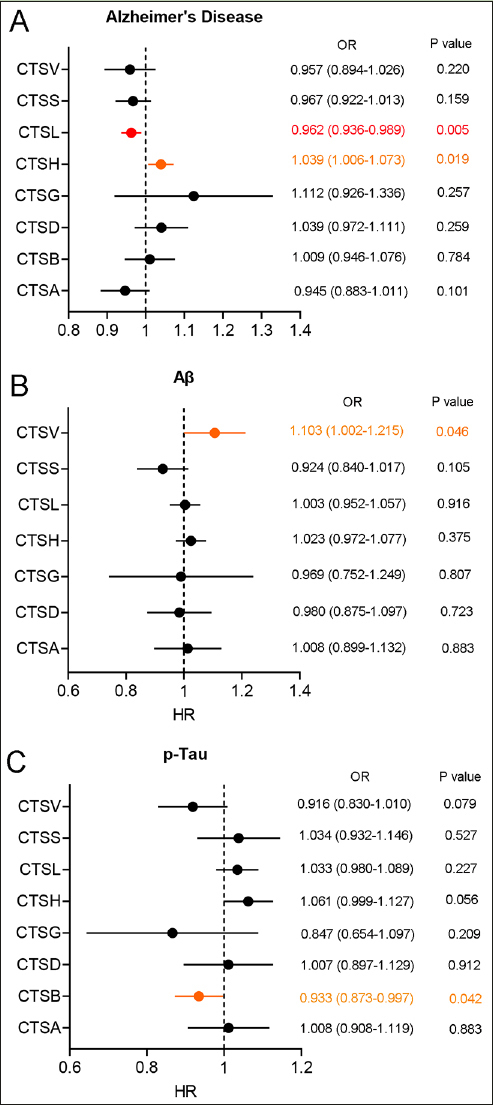
Forest plot of two-sample MR analysis for blood cathepsins on the risk of AD and CSF pathological biomarkers (A) Forest plot of two-sample MR analysis for blood cathepsins on the risk of AD through the IVW method. (B) Forest plot of two-sample MR analysis for blood cathepsins on the CSF Aβ through the IVW method. (C) Forest plot of two-sample MR analysis for blood cathepsins on the CSF p-Tau through the IVW method. OR: Odds Ratio. Highlighted in orange represented potential statistical difference before Bonferroni correction. Highlighted in red represented statistical difference after Bonferroni correction.

First of all, MR analysis was conducted to explore a causal relationship of eight cathepsins (cathepsin A, B, D, G, H, L, S, and V) on the risk of AD and related pathological features. The result of the two-sample MR analysis revealed that high level of cathepsin L (CTSL) was associated with a lower risk of AD (IVW: Odds Ratio (OR) = 0.962, 95% confidence interval (CI) = 0.936–0.989, p = 0.005) and high level of cathepsin H (CTSH) was associated with a high risk of AD (IVW: OR = 1.039, 95% CI = 1.006–1.073, p = 0.019) (Figure 1A). In addition, we found a slightly correlation of blood cathepsin V (CTSV) on increased CSF Aβ level (IVW: OR = 1.103, 95% CI = 1.002–1.215, p = 0.046) and blood cathepsin B (CTSB) on decreased CSF p-Tau level (IVW: OR = 0.933, 95% CI = 0.873–0.997, p = 0.042) (Figure 1B–C). After Bonferroni method correction, only high level of CTSL have been shown to be associated with a lower risk of AD. The Cochran’s Q statistic showed that there was heterogeneity in the MR analysis of CTSA, CTSB, CTSH, and CTSV on the risk of AD. Therefore, a random effect IVW method was used (Table S1). In addition, intercept of MR Egger regression test revealed a horizontal pleiotropy in the MR analysis of CTSB on the CSF Aβ level (Table S1). This result was not included in this study.

### Independent GWAS dataset validation and meta-analysis

**Figure 2:**
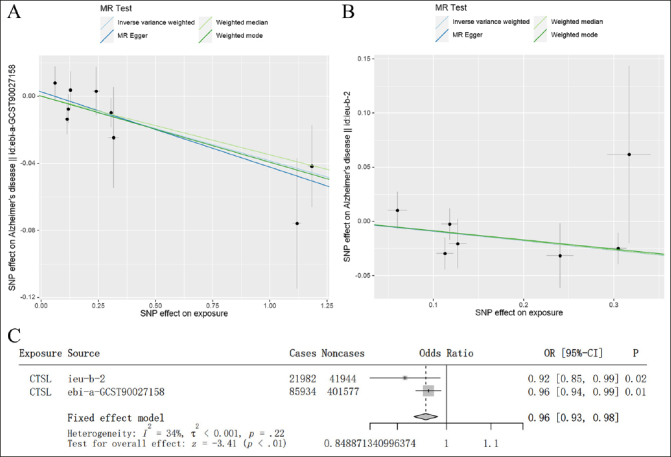
Independent GWAS dataset validation and meta-analysis confirmed the causal effect of blood CTSL on the risk of AD (A) Scatterplot of the genetic causal effect of blood CTSL on the risk of AD from the EADB. (B) Scatterplot of the genetic causal effect of blood CTSL on the risk of AD from IGAP. Each black dot indicates an SNP. Vertical and horizontal lines around each SNP show 95% confidence interval. The x-axis showed the SNPs effect on blood CTSL. The y-axis showed the SNPs effect on AD. (C) Meta-analysis of the result from the training and validation dataset.

To increase the reliability of the above results, we used independent AD GWAS data for verification. In AD GWAS data from IGAP, we found that there was a consistent result from EADB dataset that high level of CTSL was associated with a lower risk of AD (IVW: OR = 0.915, 95% CI = 0.849–0.986, p = 0.020) (Figure 2B). Similarly, the result of integrating EADB and IGAP dataset through meta-analysis were shown that a significant negative correlation between the level of blood CTSL on the risk of AD (Figure 2A–C). Therefore, consistent results were obtained from two independent AD GWAS datasets and meta-analysis.

### Participant characteristics in observational cohort

In this study, we recruited 31 Aβ− CU and 36 Aβ+ CI participants from the ADIMIC cohort to further validate the results from MR study. The demographic and clinical characteristics of each group were summarized in Table 1. The Aβ− CU group contained 13 males and 18 females with a mean age of 65.935 years. The Aβ+ CI group contained 13 males and 23 females with a mean age of 67.722 years. There was no significant difference in age (p=0.263) and gender (p=0.626) distribution between the two groups. Aβ− CU group had higher education level as well as better MMSE and MOCA score, compared to Aβ+ CI group. In addition, there was a higher SUVR of AV-45/PET (p<0.001) in Aβ+ CI group, compared to Aβ− CU group. In the detection of plasma pathological markers, Aβ+ CI group showed decreased Aβ42 (p<0.001) and Aβ42/40 level (p<0.001), companying with increased p-Tau181 (p<0.001), NFL (p<0.001) and GFAP (p<0.001) level, compared to Aβ− CU group. There was no significant difference in plasma Aβ40 (p=0.832) concentration between the two groups.

**Table 1:** Demographic data, neuropsychological testing and plasma pathological biomarkers for participants

**Characteristic**	**Aβ− CU (n=31)**	**Aβ+ CI (n=36)**	**P Value**
Age (years, mean ± SD)	65.935 ± 6.071	67.722 ± 6.772	0.263
Sex (male: female)	13:18	13:23	0.626
Education (years, mean ± SD)	12.581 ± 3.802	9.806 ± 3.823	0.004
MMSE (mean ± SD)	29.194 ± 1.108	19.972 ± 6.130	<0.001
MoCA (mean ± SD)	26.516 ± 2.755	14.000 ± 6.510	<0.001
SUVR of AV-45/PET (mean ± SD)	0.959 ± 0.043	1.436 ± 0.246	<0.001
Plasma Aβ40 (pg/ml, mean ± SD)	110.701 ± 13.343	109.972 ± 14.543	0.832
Plasma Aβ42 (pg/ml, mean ± SD)	7.595 ± 1.404	6.125 ± 1.065	<0.001
Plasma Aβ42/40 (mean±SD)	0.068 ± 0.009	0.056 ± 0.006	<0.001
Plasma p-Tau181(pg/ml, mean ± SD)	2.417 ± 0.923	4.765 ± 1.576	<0.001
Plasma NFL (pg/ml, mean ± SD)	17.430 ± 6.726	25.162 ± 6.496	<0.001
Plasma GFAP (pg/ml, mean ± SD)	110.115 ± 46.710	233.433 ± 86.080	<0.001

### Association of blood CTSL with cognitive level and plasma pathological markers in Aβ+ CI participants

We detected the expression level of CTSL in Aβ− CU and Aβ+ CI group, and found that the expression level of CTSL in Aβ+ CI group was lower than that in Aβ− CU group (Figure 4A). After that, we further explored the association of blood CTSL expression level with cognitive level plasma pathological markers in Aβ+ CI group. The result showed that blood CTSL expression level was negatively correlated with MMSE score (r=−0.538, p<0.001), MOCA (r=−0.489, p=0.004) score, plasma Aβ42 (r=−0.439, p=0.008) and Aβ42/40 (r=−0.444, p=0.007) level in Aβ+ CI group (Figure 4B–D, F). There was no significant correlation between the expression level of blood CTSL and plasma Aβ40 (r=−0.131, p=0.447), p-Tau181 (r=0.046, p=0.790), NFL (r=0.123, p=0.474), and GFAP (r=0.109, p=0.527) (Figure 4E, G-I).

**Figure 3:**
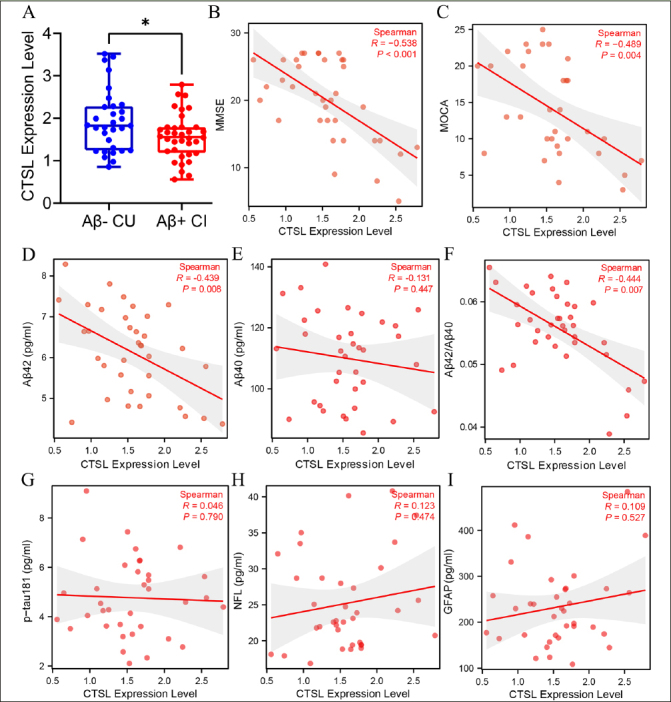
Detection of blood CTSL expression level and correlation analysis with cognitive and pathological markers (A) The difference analysis of CTSL expression level between Aβ− CU and Aβ+ CI group. Data was analyzed using two-tailed Student’s t test. *P <0.05. (B-I) Spearman correlation analysis of blood CTSL expression level with MMSE score, MOCA score, plasma Aβ42, Aβ40, Aβ42/40, p-Tau181, NFL, and GFAP level in Aβ+ CI group. MMSE: Mini-mental state examination, MOCA: Montreal cognitive assessment, CU: cognitive unimpaired, CI: cognitive impaired.

**Figure 4:**
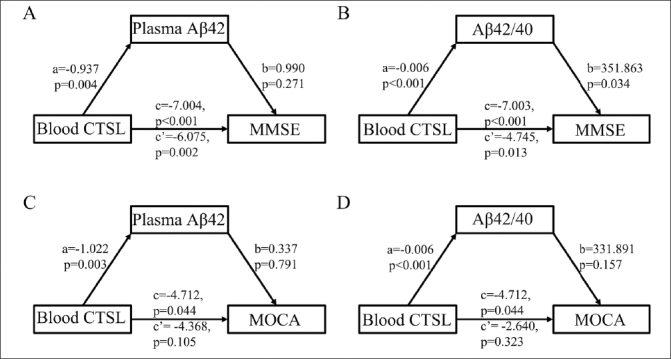
Mediated analysis of CTSL expression level, cognitive level and plasma Aβ pathologic markers in Aβ+ CI group (A) Mediating effect of plasma Aβ42 on association between CTSL expression level and MMSE score in Aβ+ CI group. (B) Mediating effect of plasma Aβ42/Aβ40 on association between CTSL expression level and MMSE score in Aβ+ CI group. (C) Mediating effect of plasma Aβ42 on association between CTSL expression level and MOCA score in Aβ+ CI group. (D) Mediating effect of plasma Aβ42/Aβ40 on association between CTSL expression level and MOCA score in Aβ+ CI group. MMSE: Mini-mental state examination, MOCA: Montreal cognitive assessment, CI: cognitive impaired.

### Plasma Aβ42/40 level was the key mediator in the association between blood CTSL and cognitive level in Aβ+ CI group

We performed a mediation analysis to explore the correlation among blood CTSL expression level, plasma Aβ pathological markers and cognitive level in Aβ+ CI group. The results showed that there was no mediating effect between blood CTSL expression level, plasma Aβ42 and MMSE scors (Figure 4A). The plasma Aβ42/Aβ40 level (mediating ratio = 32.2%) mediated the association between blood CTSL expression level and MMSE score with statistical difference in Aβ+ CI participants (Figure 4B). In addition, there was no mediating effect between blood CTSL expression level, plasma Aβ pathological markers and MOCA scores (Figure 4C–D).

## Discussion

Alzheimer’s disease, the main type of dementia, involves in complex pathophysiological processes, including abnormal lysosomes function (33). Cathepsins are the predominant proteases responsible for the degradation of diverse substrates in the endo-lysosomal system (34). Previous preclinical and clinical studies have elucidated association between several cathepsins and AD (34). However, there was still a lack of systematic study on the causal association between cathepsins and AD.

In this study, we aimed to explore the association of blood cathepsins with the risk of AD and related pathological features through observational cohort and MR study. Using MR method, we observed that high level of CTSL was associated with a lower risk of AD through Bonferroni correction and independent GWAS data verification. In observational cohort, we found that the blood CTSL expression level in Aβ+ CI group was lower than that in Aβ− CU group. In 2022, Islam et al. showed that the expression level of CTSL in the hippocampal neuron of AD patients was significantly higher than that of the control group (35). The above result was contrary to our result, which may be related to the difference in the expression of CTSL in different cell type. More important, our result revealed that blood CTSL expression level was negatively correlated with MMSE score, MOCA score, plasma Aβ42 and Aβ42/40 level in Aβ+ CI group. Mediation analysis showed that plasma Aβ42/40 level was the key mediator in the association between blood CTSL and MMSE score in Aβ+ CI participants. In previous preclinical studies, researchers demonstrated that the level of Aβ42 can be reduced by pharmacological or genetic knockout of Ctsl (35). Ctsl affected the Aβ pathology through degradation of C-terminal fragments of the amyloid precursor protein (APP) and β-secretase in the APP processing (36–38). These results may explain the effect of CTSL on MMSE score mediated by plasma Aβ42/40 in this study.

In addition, we found that higher CTSH level were associated with an increased risk of AD before Bonferroni correction. In a large-scale GWAS study, *CTSH* gene was identified as a new AD-associated risk gene in European populations (20). A recent study identified rs2289702 in *CTSH* gene as a protective functional variant of AD in Han Chinese population (12). Similarly, proteome-wide association study (PWAS) revealed the relationship between *CTSH* gene and AD (39). At the transcriptional level, there was significantly increased CTSH expression level in both AD patients and animal models (12). Knocking out *CTSH* gene in human microglia can significantly improve the phagocytosis of Aβ peptides (12). All these studies indicated the genetic association and potential role of CTSH gene in AD.

In AD related pathological biomarkers, our result revealed that high blood CTSV level was slightly associated with increased Aβ level in CSF and high blood CTSB level was slightly associated with decreased p-Tau level in CSF before Bonferroni correction. Up to now, there was a lack of study on the correlation between CTSV and AD and pathological markers. On the contrary, the correlation between CTSB and AD has been widely reported. Numerous of studies reported that CTSB expression level was significantly increased in serum, plasma and CSF of AD patients (40). In AD mouse, cathepsin B (CatB) knockout can reduce memory deficits and Aβ plaque load (40). However, adeno associated virus (AAV) mediated hippocampal overexpression of CatB can improve cognitive impairment and reduce Aβ load in APP/PS1 mice (41). Therefore, the role of CTSB in the occurrence and progression of AD was complex. Future study should focus on the functional difference of CTSB in different cell type.

The study has several limitations. First of all, this study included an observational cohort with a small sample size. Secondly, the molecular mechanism of cathepsins involvement in AD was not thoroughly clarified. In the future study, we will further focus on the role and mechanism of cathepsins in different cell types of AD. In summary, this study comprehensively analyzed the association of cathepsins with the risk of AD and pathological markers through MR and observational cohort study. This study provided an important basis for clarifying the importance of cathepsins in the occurrence and progression of AD and its potential regulatory mechanism.

## Electronic supplementary material

Supplementary material, approximately 490 KB.

## Abbreviations

AD: Alzheimer disease
Aβ: Amyloid-β
NFTs: neurofibrillary tangles
BBB: blood-brain barrier
MR: mendelian randomization
IVs: instrumental variables
CSF: cerebrospinal fluid
GWAS: genome-wide association data
GFAP: glial fibrillary acidic protein
NFL: neurofilament light
ADIMIC: Alzheimer’s disease Immunization and Microbiota Initiative study Cohort
EADB: European Alzheimer & Dementia Biobank
IGAP: International Genomics of Alzheimer’s Project
MAF: minor allele requency
IVW: inverse-variance weighted
MCI: mild cognitive impairment
NIA-AA: National Institute on Aging and Alzheimer’s Association
CI: cognitive impaired
CU: cognitive unimpaired
MMSE: Mini-Mental State Examination
MoCA: Montreal Cognitive Assessment
CDR: Clinical dementia rating
AVLT: Auditory Verbal Learning Test
MPRAGE: magnetisation prepared-rapid gradient echo
FLAIR: fluid-attenuated inversion recovery
Simoa: single molecule array
CTSL: cathepsin L
CTSH: cathepsin H
CTSV: cathepsin V
CTSB: cathepsin B
OR: Odds Ratio
APP: amyloid precursor protein

## Notes

*Ethics declarations:* The dataset from the GWAS studies have been approved by the ethics committee. The cohort study protocol was approved by the Review Committee Research Committee of Ruijin Hospital, Shanghai Jiao Tong University School of Medicine (approval number: 2021–46). All participants provide written informed consent prior to participation.
