# Supplementary material for: Blood Cathepsins on the Risk of Alzheimer’s Disease and Related Pathological Biomarkers: Results from Observational Cohort and Mendelian Randomization Study
Source: J Prev Alzheimers Dis. 2024 Jun 11;11(6):1834–42. doi: 10.14283/jpad.2024.107 (PMC11573867; doi:10.14283/jpad.2024.107)

**Table S1:** **Heterogeneity and horizontal pleiotropy analysis of two-sample MR study.**

| Exposure | Outcome | Cochran's Q | P Value | MR-egger intercept | P Value |
| --- | --- | --- | --- | --- | --- |
| CTSA | AD | 32.659 | 0.008 | -0.004 | 0.537 |
| CTSB |  | 31.040 | 0.001 | 0.017 | 0.147 |
| CTSD |  | 9.865 | 0.196 | -0.0002 | 0.985 |
| CTSG |  | 5.513 | 0.138 | 0.009 | 0.749 |
| CTSH |  | 32.255 | 0.041 | 0.002 | 0.723 |
| CTSL |  | 4.560 | 0.803 | 0.003 | 0.643 |
| CTSS |  | 8.194 | 0.831 | -0.001 | 0.853 |
| CTSV |  | 29.708 | 0.020 | -0.005 | 0.557 |
| CTSA | CSF Aβ | 25.816 | 0.078 | -0.007 | 0.566 |
| CTSB |  | 11.828 | 0.460 | -0.028 | 0.035 |
| CTSD |  | 4.373 | 0.822 | -0.001 | 0.957 |
| CTSG |  | 3.220 | 0.522 | -0.043 | 0.235 |
| CTSH |  | 19.859 | 0.468 | 0.002 | 0.803 |
| CTSL |  | 2.801 | 0.903 | -0.001 | 0.905 |
| CTSS |  | 19.388 | 0.249 | 0.011 | 0.199 |
| CTSV |  | 8.693 | 0.949 | 0.008 | 0.490 |
| CTSA | CSF pTau | 20.602 | 0.245 | -0.006 | 0.581 |
| CTSB |  | 12.859 | 0.379 | 0.015 | 0.260 |
| CTSD |  | 8.0376 | 0.430 | -0.009 | 0.548 |
| CTSG |  | 3.471 | 0.482 | -0.020 | 0.539 |
| CTSH |  | 27.489 | 0.122 | 0.002 | 0.874 |
| CTSL |  | 6.638 | 0.468 | -0.009 | 0.454 |
| CTSS |  | 21.670 | 0.154 | 0.003 | 0.740 |

**Figure S1: Study design** **flow chart.**


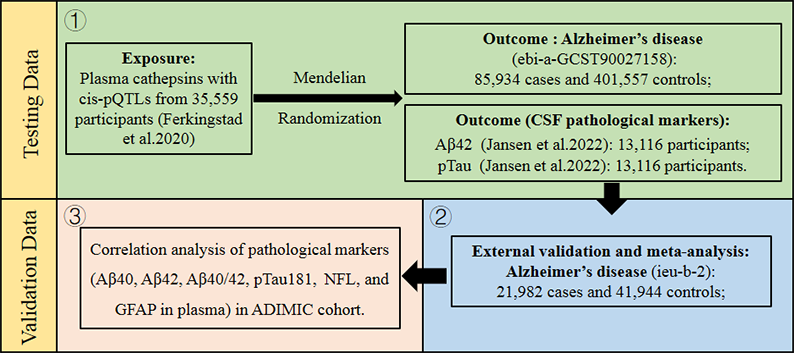

Supplement: Supplementary file 1 — Supplementary material, approximately 490 KB. [file 42414_2024_355_MOESM1_ESM.docx]
